# Patients’ experiences of chronic pain and sleep as a vicious circle: a qualitative explorative study from a pain rehabilitation setting

**DOI:** 10.3389/fpain.2026.1869706

**Published:** 2026-07-30

**Authors:** Andrea Hållstam, Charlotte Angelhoff, Linda Gellerstedt

**Affiliations:** 1 Department of Clinical Sciences, Danderyds Hospital, Karolinska Institutet, Stockholm, Sweden; 2 Department of Health, Medicine and Caring Sciences, Linkopings Universitet, Linköping, Sweden; 3 Allergy Center, Universitetssjukhuset I Linkoping, Linköping, Sweden; 4 Department of Neurobiology, Care Sciences and Society, Karolinska Institutet, Stockholm, Sweden

**Keywords:** bidirectional relationship, chronic pain, insomnia, IPRP, long-lasting pain, sleep, sleep-ergonomic

## Abstract

**Objective:**

Chronic pain and sleep disturbances are common, interrelated conditions that substantially affect quality of life. Patients participating in interdisciplinary chronic pain rehabilitation programs frequently report sleep problems, yet patients’ own experiences of sleep within this context remain insufficiently explored. The aim of this study was to describe experiences of sleep and pain among patients with diverse chronic pain conditions who have undergone an interdisciplinary pain rehabilitation program.

**Methods:**

An explorative qualitative descriptive design was applied. Data were collected through focus group interviews and individual interviews with patients participating in an interdisciplinary pain rehabilitation program. The data were analysed using reflexive thematic analysis. Reporting followed the Consolidated Criteria for Reporting Qualitative Research (COREQ).

**Results:**

The bidirectional relationship between pain and sleep was a central aspect of participants’ daily lives. The overarching theme, *A vicious circle with two inseparable conditions*, reflects how participants experienced pain and sleep disturbances as closely intertwined and difficult to distinguish. Three interconnected themes further described these experiences: *Struggling with the interplay between pain and sleep; Striving for balance and meaning in daily life;* and *Needing understanding, support, and self*-*compassion*. Participants described fragmented sleep, persistent fatigue, and continuous disruption of rest by pain, alongside ongoing efforts to manage everyday life under these conditions. An emotional burden was evident, characterized by self-doubt and questioning the legitimacy of their suffering.

**Conclusion:**

The findings illustrate how chronic pain and sleep disturbances coexist in a mutually reinforcing vicious cycle that significantly impacts quality of life. By elucidating the practical, emotional, and social dimensions of these experiences, the study contributes to an extended understanding of chronic pain from a patient perspective. The results underscore the importance of integrating sleep hygiene practices, sleep ergonomics, and holistic, patient-centred approaches within interdisciplinary chronic pain rehabilitation programs.

## Summary

- This study illustrates how chronic pain and sleep disorders coexist, creating a vicious cycle significantly impacting quality of life. The descriptions of practical, emotional, and social impacts contribute to an extended understanding for individuals living with chronic pain.
- It provides novel insights into patient experiences across diverse pain conditions following interdisciplinary rehabilitation, underscoring the need for enhanced focus on sleep hygiene and ergonomics.
- The study offers new and extended insights of experiences of sleep and pain among patients with diverse pain conditions who have undergone an interdisciplinary pain rehabilitation program. Findings support a holistic approach to chronic pain rehabilitation, emphasizing the integration of sleep-related interventions.

## Introduction

Sleep and pain are complex neurological processes, crucial for human survival. Chronic pain, insomnia and sleep difficulties fundamentally affect a person's life. Thus, when coexisting, the burden is multiplied (1). In recent years, the connection between pain and sleep has garnered increased interest in both basic and clinical research. Both conditions are influenced by biological, psychological, and social factors, and their relationship is bidirectional (2–4).

The psychological processes in chronic pain and sleep disorders include mood and affect, dysfunctional beliefs as well as coping strategies (5, 6). Social factors important for chronic pain and sleep disorders are found in both personal and environmental circumstances (6, 7). Although the prevalence of sleep disorders in the chronic pain population varies, the magnitude, i.e., the majority of individuals, have sleep difficulties that remain similar across diverse pain conditions (8). This includes patients with chronic musculoskeletal pain (9), endometriosis (10), diabetic neuropathic pain (11) and patients participating in interdisciplinary pain rehabilitation programs (IPRP) (12, 13). Long-term sleep disorders have been shown to significantly contribute to the development of chronic spinal pain in individuals compared to those without disrupted sleep (14). Pain leading to insomnia has also been found (15, 16). Sleep difficulties are moreover inversely associated to the probability of recovery from chronic low back pain (14, 17).

Based on the biopsychosocial model, the treatment of chronic pain or insomnia is primarily nonpharmacological and preferably treated with a team-based, multimodal approach when coming to chronic pain (18, 19). In IPRP, the overall aim is to improve function, activity and participation, thus quality of life. This is primarily achieved through psychological methods, physiotherapy, and patient education (2). To address sleep difficulties, interventions in IPRP can also include sleep hygiene practices (20) and sleep ergonomics (21). Results from IPRP show improvement in insomnia, especially for individuals with chronic pain who had more sever insomnia before starting the programme (12); however, other studies are inconsequential about the outcome of the pain programme on sleep difficulties (22–24).

Research on individuals' experiences of chronic pain and sleep disturbances primarily focuses on those with nociplastic pain (25). For example, patients with fibromyalgia experienced poor sleep quality with a profound impact on health and other symptoms, demonstrating a bidirectional connection between pain and sleep. These individuals expressed feeling insufficiently equipped with strategies to cope with poor sleep or its consequences (25). Insufficient sleep was defined as difficulties falling asleep and frequent awakenings during the night and thus consequences for daytime life such as fatigue, stiffness and cognitive impairment. Further consequences were impaired daily activity and eating behaviours (25, 26). Strategies to support sleep mainly included the use of medication, maintaining regular sleep schedules, using practical aids, or considering leaving the bed. However, most of these strategies have proven to be insufficient. In the same study, the need for enhanced understanding of effective sleep strategies for individuals with fibromyalgia was highlighted (25). Further mindfulness was found to be the most effective community-based intervention for improving sleep in patients with fibromyalgia, offering benefits such as greater patient independence from health care, potential for widespread dissemination, and reduced costs (27).

Sleep disturbances are present in many other pain conditions besides fibromyalgia and in individuals participating in IPRP. However, their experiences of what interferes with salutary sleep or the significance of IPRP in managing sleep difficulties have not yet been described. To further develop IPRP and to better meet individuals' needs, extended knowledge of their experiences and what truly matters to them is essential. This study therefore aimed to describe experiences of sleep and pain among patients with diverse pain conditions who have undergone an IPRP.

## Methods

### Design

An explorative qualitative descriptive design (28) was applied and conducted through focus groups interviews (FGI) and individual interviews and reported according to the 32-item checklist, consolidated criteria for reporting qualitative research (COREQ) (29).

### Context and recruitment of participants

The study was performed at a highly specialised rehabilitation clinic in Stockholm, Sweden. The pain rehabilitation process starts when referred patients meet a team comprising a nurse, physician, psychologist, physiotherapist, occupational therapist, and social worker to assess patient needs and resources but also hindrances. During a team conference, patients are discussed, and suitable rehabilitation plans are developed for completion in the clinic. If this is not considered appropriate, they are sent back to the referring physician. The IPRP consists of a three-week individual digital introduction programme, where patients take part in video films with a follow-up by team members. The following on-site IPRP programme consists of about two days of interventions per week, for seven weeks in groups of four to eight patients. The programme includes psychological, physiotherapeutic, and occupational therapy interventions as well as education about pain, health, drugs and information from the social worker, physician and nurse. Furthermore, sleep theory, sleep hygiene, and sleep ergonomics are integrated into the IPRP. Interventions may involve education regarding sleep-promoting behaviours, optimisation of the sleep environment, individual assessment of mattress and pillow suitability, and evaluation of alternative sleeping positions.

All patients undergoing group-based IPRP at the clinic from January 2022 to April 2024 were invited to participate in the study both verbally and in writing through a purposive sampling. Information was provided during the group-based IPRP along with a preliminary interview date. The inclusion criteria were the same as those for inclusion in the IPRP: chronic pain affecting daily life and previous pain treatment in primary care with limited success. Exclusion criteria were underlying disease not fully investigated or treated, drug use disturbing the rehabilitation process, need for a language interpreter, and factors affecting the ability to participate in group-based IPRP.

## Data collection

Thirty-four individuals were invited and scheduled to participate in a focus group interview by the study's first author, who did not participate in the interviews. Of these, 27 provided informed consent, and 18 ultimately participated in either a focus group interview (FGI) or an individual interview. As participants were living with chronic pain that affected their daily functioning, periods of severe pain, along with related symptoms such as fatigue and reduced mobility, sometimes hindered their ability to take part in scheduled data collection activities. Consequently, some participants cancelled their planned participation either the day before or on the day of the scheduled FGI. No individuals withdrew during or after the interviews. Although focus groups were the primary method of data collection, individual interviews were conducted when participants who had expressed an interest in the study were unable to attend a scheduled focus group or when other planned participants did not attend. Offering individual interviews ensured that willing participants were not excluded and contributed to informational redundancy across the dataset. In total, 18 participants were interviewed, either in an FGI (*n* = 12) or in an individual interview (*n* = 6) (Table 1).

**Table 1:** Demographics of participants in the FGI and individual interviews.

Characteristic	In total	FGI 1	FGI 2*	FGI 3*	FGI 4	FGI 5*	FGI 6*	FGI 7*	FGI 8*	FGI 9
Participants (n) (invited)	18 (34)	6 (7)	1 (4)	1 (7)	3 (4)	1 (3)	1 (2)	1 (2)	1 (2)	3 (3)
Women (n)	15	6	1	0	3	1	1	1	1	1
Men (n)	3	0	0	1	0	0	0	0	0	2
Age (m)		54	44	55	45	28	54	38	56	56
Interview setting		Clinic	Clinic	Clinc	Clinic	Clinic	Clinic	Digital	Clinic	Clinic
Interview time (min)	7,498	84	42	48	97	56	46	44	70	81

* Focus groups (FGI) nr 2, 3, 5, 6, 7 and 8 were performed as individual interviews due to withdrawals from scheduled FGI.

The participants represented a range of pain diagnoses, such as hypermobility spectrum disorders/Ehlers-Danlos syndrome (*n* = 6), neuropathic pain (*n* = 5), fibromyalgia (*n* = 4), and visceral pain (*n* = 3). Three FGIs were held face-to-face at the clinic, facilitated by a moderator (LG) and an observer (CA), who took field notes and occasionally contributed to the discussion. Six individual interviews were conducted either face-to-face or digitally by one of the authors (LG or CA). A topic guide developed within the research group was used for both FGIs and individual interviews (Table 2). All interviews were audio-recorded with consent, and none of the two female researchers who conducted the interviews were employed by the clinic as part of the IPRP, nor had any prior relationship with the participants.

**Table 2:** Interview guide for FGI and individual interviews.

Questions
- What does sleep mean to you?
- What function does sleep serve for you? Can you give an example?
- In what way does your sleep affect your chronic pain? - If so, can you give an example of how? - In what way does chronic pain affect your sleep? - If so, can you give an example of how?
- What are your thoughts on sleep and its impact on quality of life? - Can you give an example?
- What are your thoughts on pain and its impact on quality of life? - Can you give an example?
- What helps you manage sleep and pain? - Do you have any strategies or tools that work for both sleep and pain?
- What challenges do you face in managing sleep and pain?
- How has your participation in the rehabilitation programme affected your pain and sleep? - Can you give an example?
- Do you have any requests for additional or different aspects of sleep that should have been included in the IPRP?
- Do you have anything else to add or want to share about sleep and pain?
- Do you have anything else you would like to share about your participation in the IPRP?

## Data analysis

The analysis commenced after all interviews had been conducted and the audio-recorded data were transcribed verbatim and pseudonymised. The analysis was undertaken by the three female authors, all registered nurses with PhD qualifications, and was performed manually (i.e., no qualitative software programme was used). LG and CA have extensive clinical and research experience in sleep and health. AH has extensive clinical experience in pain management and pain rehabilitation, with a research focus on pain and pain management.

Data were analysed using reflexive thematic analysis (30) with an inductive approach for its ability to capture diverse experiences, providing flexibility (31, 32). The process of analysis followed the described six-phased approach (31). Firstly, all authors repeatedly read and listened to the transcribed interviews to familiarise themselves with the data. In phase 2, the authors independently identified segments of data and then collaboratively coded the segments. During the coding process, the authors actively engaged in discussions and collectively reviewed the codes multiple times. In phase 3, the codes were grouped in blocks of preliminary themes, pulling together patterns of meaning. In phase four, the completed coding and preliminary themes were discussed, revised, and developed by all authors. In phase five, the interpretations of findings and the thematic structure were discussed and refined by all authors until one main theme and three themes were formulated. Throughout the data collection and analysis process, the researcher reflected on how their professional background and prior experience in clinical care might influence both the interview process and the interpretation of data. To mitigate this potential impact, interviews were conducted using open-ended questions and a neutral approach, allowing participants to articulate their experiences in their own words. Furthermore, the interviews were conducted by two of the authors, neither of whom had prior clinical experience in pain rehabilitation. During the analysis phase, reflexivity was enhanced through continuous team discussions and iterative reviews of emerging themes, thereby reducing the influence of individual preconceptions on the interpretation.

**Figure 1:**
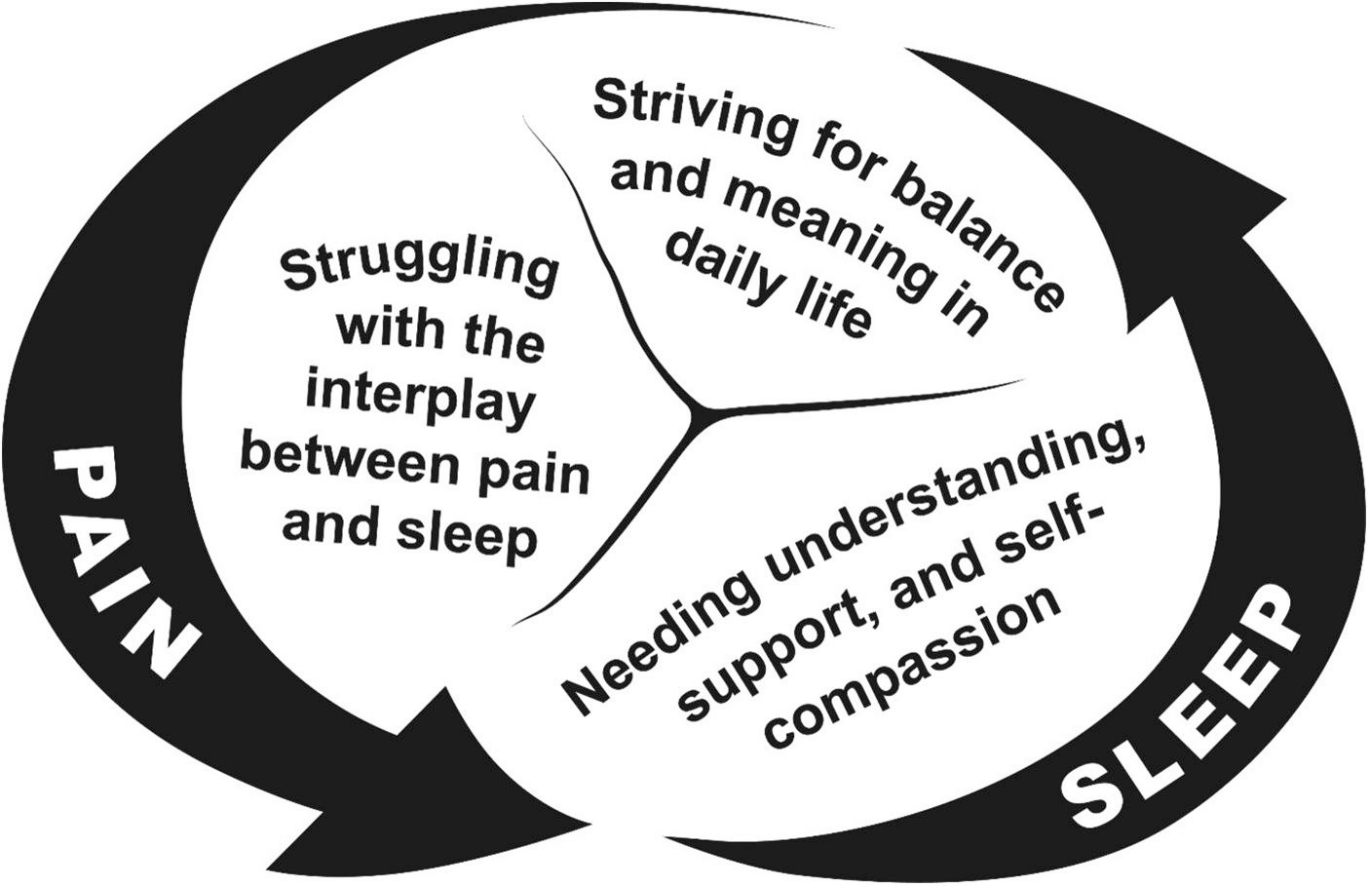
Main theme and subthemes describing participants' experiences of living with pain and sleep difficulties. The circular model illustrates the reciprocal relationship between pain and sleep and comprises three interconnected subthemes: struggling with the interplay between pain and sleep, striving for balance and meaning in daily life, and needing understanding, support, and self-compassion.

After the fifth phase, four participants from two of the conducted FGIs were invited by phone to participate in a member checking (MC) (33) as part of patient contribution and a validation of the analysis. The preliminary findings and five reflection questions were sent to the participants one week before the member checking (Table 3). Three of four invited participants gave consent to participate in the member checking as a digital FGI, and one participant consented but chose to give a written individual response. The member check was performed as a digital FGI, and the participants were asked to discuss their views of the findings based on the reflective questions. One author (LG) was moderating, and two authors (AH and CA) were observers during the MC, which lasted for 60 min. Reflections on the preliminary findings were consistent across participants involved in the member checking, with no discrepancies identified between those participating in the digital member checking and the individual who provided written feedback. None of the four participants wished for anything to be removed or edited. They expressed how they recognised themselves in the text and that the main theme captured their lived situation. The findings were perceived as congruent with their intended expressions and the themes related to their experiences, but minor modifications of Figure 1 were suggested. Additionally, in phase six, all authors contributed to the writing of the findings, and Figure 1 was revised accordingly to reflections during the MC. Each step of the analysis process was characterised by flexibility and repetitive verification in relation to the transcribed interviews, codes, and formulated themes. Regarding informational redundancy (34), eighteen participants were considered sufficient to address the research question, particularly as the study population comprised a vulnerable group. The use of member checking further indicated that informational redundancy had been achieved, suggesting that data saturation had been reached, as the process did not generate new insights or prompt substantive revisions to the findings.

**Table 3:** Reflective questions for member checking.

Questions
- After reading the findings, what are your general thoughts?
- If there is anything you would like removed? What would that be, and why?
- How accurately do you feel the findings captured your thoughts/experiences?
- What could be added to the findings to capture your experiences better?
- How is the figure illustrating your experiences?

## Ethical considerations

The study was approved by the Swedish Ethical Review Authority (Dnr 2022-06986-01 and Dnr 2024-02126-02) and performed in accordance with the Helsinki Declaration (35). Before inclusion, all participants were informed, both verbally and in written form, about the study, and all participants signed an informed consent form. The findings are presented in a way that ensures that no participant can be identified.

## Findings

The findings reveal a complex interplay between pain and sleep disturbances, described in three themes (Figure 1): *Struggling with the interplay between pain and sleep* focuses on fragmented sleep and the “snowball effect” of pain and restlessness; *Striving for balance and meaning in daily life* highlights efforts to adapt activities and find purpose despite challenges of pain and sleep disturbances and fatigue; and *Needing understanding, support, and self-compassion* addresses the emotional toll of living with invisible conditions, and unseen symptoms, emphasising the value of support, self-compassion, and societal understanding.

## A vicious circle with two inseparable conditions (main theme)

The main theme encompasses the intricate interplay between pain and sleep disturbances, described as a vicious cycle where pain and sleep were hard to separate. Pain exacerbates sleep difficulties, while poor sleep intensifies pain, creating a cycle of mutual influence leading to falling asleep itself becoming a source of anxiety. The unreachable need for rest imposed a mental burden, worsening their suffering. This cycle profoundly affected quality of life and daily functioning and reduced the pursuit of balancing and managing life. Furthermore, the importance of self-compassion, support, and understanding when living with an invisible condition was emphasised.

## Struggling with the interplay between pain and sleep (theme 1)

This theme describes the challenges of sleeping difficulties and experiences of fragmented sleep as well as how pain interrupted rest and sleep. Sleep difficulties intensified the experience of pain, and the pain, in turn, affected the ability to sleep.

“So, you lie there, and it's not just that your mind is racing with thoughts, but you also have the physical pain, it just hurts. It doesn't matter what position I take, it hurts anyway, and of course, laying there makes it even worse, like a snowball effect”. (FGI 2)

The interplay between pain and sleep was described as profoundly impacting quality of life. The participants described feeling trapped by the limitations that their pain and sleep issues caused. Some participants explained how they tried to go to bed earlier to get more sleep, but this instead led to more pain as issues such as dislocated joints made it difficult to catch up and rest. They described feeling constrained by their symptoms and struggling to achieve restorative sleep to allow their bodies to recover properly. On the other side, some participants described sleep as a kind of escape and temporary refuge from pain. Most participants described their sleep as fragmented and that they never experienced enough hours of uninterrupted sleep. For some, this led to sleep avoidance due to the pain it could trigger:

“The pain wakes me up, then I’m awake and unable to relax. I become afraid of falling asleep again, and that creates a vicious cycle”. (FGI 1, participant no. 4)

Several participants expressed frustration at how these coexisting conditions led to the attempt to get sleep becoming a source of anxiety. Stress was perceived as a common consequence of sleep disturbances, and it was compounded by the awareness of the need for sleep, which increased mental strain. Night-time stress was also often linked to financial concerns, particularly related to insurance matters, creating a paradox where the body's need for rest exacerbated suffering. They perceived their sleep as very poor, and instead of recovery, they felt even more tired after sleep. Some described constantly feeling on the edge, never waking up rested.

“I never wake up rested; I’m almost more tired when I wake up because I've slept so poorly. It feels like it would have been better if I hadn't slept at all”. (FGI 2)

The participants shared some of their strategies to manage sleep difficulties. Some practised mindfulness or relaxation techniques learned during the programme. Other strategies for rest were to sleep on the sofa or take the moments of possibility to fall asleep during the daytime. Most participants applied sleep ergonomics, for example, using appropriate pillows and positioning to relieve pressure on the neck and back, enhancing sleep conditions. Practical aids, obtained during the programme, such as sliding sheets for easier movement and turning, were mentioned as they improved both sleep and pain. Another strategy was accepting being awake during the nights. Some of their efforts were helpful; however, sleep was mostly not experienced as sufficiently restorative, and the situation after the IPRP was still described as a constant struggle.

## Striving for balance and meaning in daily life (theme 2)

This theme illustrates the participants’ continuous efforts to attain a sense of balance in everyday life, as they strive to derive meaning and sustain daily functioning despite the interrelated challenges of chronic pain, sleep disturbances, and fatigue. The importance of finding balance was described as crucial for stability, and the importance of meaning was emphasised as they expressed how they sought not just survival but a fulfilling life and finding value in the small victories of every day. While enjoyable activities brought joy, they often also led to exhaustion and extended pain. Daily activities at work or with the family were voiced as difficult to realise, as the participants never could predict their daily condition in terms of energy or cognitive function.

“I can't just tell my child, like, ‘No, sorry, you must get dressed yourself, you have to go to school on your own,’ just because I don’t have the energy, because I can’t get going in the morning, or because I slept poorly”. (FGI 4, participant no. 2)

While enjoyable, activities could be energy-draining and cause side effects lasting several days. Nonetheless, these activities were crucial for fostering social connections and enhancing meaning. Participants emphasised the importance of engaging in manageable activities that replenish energy.

“You can look forward to something really nice in the evening or an activity where you think ‘this is going to be fun,’ and then as the hours approach, you start feeling like this isn't going to work, it won't happen.. unfortunately, many, many times I've pushed through, and then I'm exhausted for several days. I've also tried to work on this a lot, maybe just be there for an hour, then go home”. (FGI 9, participant no. 1)

One participant expressed that a key strategy for achieving balance in daily life was adjusting activity patterns. Participants scheduled short breaks, walks, and exercise throughout the day, as well as planned periods for rest, such as napping after work or other activities. Adaptations, like using a chair while cooking, were common and some used mindfulness, breathing techniques, and relaxation exercises for recovery, but not everyone found this helpful. Another strategy involved breaking tasks into smaller segments instead of completing them all at once. Although this advice was provided during the rehabilitation programme, it was difficult to implement due to participants' tendency to “push through” and finish tasks quickly.

“I think I have gotten better at taking breaks. One strategy that has worked well is forcing myself to take breaks while I'm working on something. I don't have to finish what I'm doing immediately; I can pause for a bit and then resume later”. (FGI 5)

Work-life adjustments were described as essential, with supportive employers and flexible hours enabling adaptation to daily conditions. Some days, pain made it impossible to go to work, and part-time sick leave was common. Others received workplace modifications to continue working and even retrained for a more suitable job. This could involve experiencing too much pain to get out of bed or continuing to struggle despite fatigue. The need to listen to the body vs. ignoring it was described as a balancing act.

“For my part, I have been able to significantly reduce my pain by slowing down the pace of my life, cutting back on activities, and when I do that, I experience less pain and sleep better”. (FGI 1, participant no. 6)

## Needing understanding, support, and self-compassion (theme 3)

This theme presents the emotional burden of living with coexisting pain and sleep difficulties as well as fatigue, including self-doubt about their legitimacy. The burden was compounded by misunderstandings and scepticism from others due to the absence of visible symptoms. Participants underlined the importance of support from both loved ones and the healthcare system. The invisibility of the highlighted conditions often led to disbelief and misjudgement, even from healthcare professionals, reinforcing participants' isolation and frustration. Due to the lack of visible signs, several participants expressed that their pain, along with its associated limitations and consequences, was often questioned. One woman recalled being challenged for using a disabled seat on public transport.

“I notice that when I take the subway or bus, I'm often so tired that I really need to sit down. However, how do I ask someone to move and stand up so I can sit? Because my pain isn't visible—I don't have a cane or a walker that clearly shows I need it”. (FGI 1, participant no. 1)

The lack of societal understanding and visible signs of distress heightened their emotional burden. One man expressed frustration at being told to “learn to live with it” as his pain remained severe despite years of suffering. This absence of understanding led to frustration, guilt, and reinforced self-doubt. Many struggled to appear “normal” while battling internal challenges. One participant described maintaining a facade despite physical exhaustion to avoid scrutiny or pity.

“But in the end, you don't know what the underlying cause is, so what should I focus on? Should I just trim the branches neatly, so it doesn’t show on the outside? I put on some mascara and take a few pills to keep going, but in the end, it's just a facade, and inside.. my soul feels worn out”. (FGI 4, participant no. 2)

The emotional cost of not being believed extended to their social lives. They shared the difficulty of disappointing loved ones and friends, especially when cancelling plans or bowing out of social events. The guilt of letting others down was compounded by their frustration at not being able to participate as they once had. One woman described the sadness of constantly letting her friends down by cancelling planned meetups.

“Even with friends you’ve made plans with you say, “No, I can’t make it today”, and then the same thing happens a second and third time. Eventually, you can see their disappointment because they really want you to join for coffee or do something together.. There's also a sadness for yourself and a sense of disappointment for them as well”. (FGI 1, participant no. 5)

For most of the participants, connecting with others who shared similar experiences during the IPRP-programme was crucial. Engaging with peers who understood the complexities of pain and sleep difficulties provided validation and alleviated emotional isolation. They highlighted the significant role of the rehabilitation programme, which not only offered valuable insights into pain and sleep management but also facilitated interactions with others facing similar challenges. The combination of educational resources and peer support empowered them to better manage their condition. Additionally, they emphasised the importance of mutual trust within the group, particularly when addressing sensitive topics. The limited number of participants in each group was moreover expressed, contributing to an increased sense of belonging, and relationships were developed.

Acquiring knowledge about their condition was crucial for participants in managing pain and sleep issues. Many reported that a deeper understanding of their pain provided a sense of control and facilitated acceptance of their situation, despite the inability to fully overcome it. Accepting the need to ask for and receive help with tasks previously managed independently was challenging, but it enabled participants to move forward and adapt to life. The need for understanding and support was followed by the necessity of self-compassion and acceptance. The process required not only seeking help from others but also learning to forgive oneself for not always being able to meet the expectations of oneself, society or loved ones.

“You doubt yourself a bit sometimes… like, ‘I can’t do this or that’… but it's important not to belittle yourself just because you are not able to do something”. (FGI 8)

## Discussion

The present study provides new insights into patient experiences, with a focus on sleep, after a group-based rehabilitation programme for chronic pain. While previous research has extensively documented challenges faced by individuals' suffering, this study highlights several novel aspects that contribute to understanding living with pain, sleep difficulties, and fatigue as a unified experience.

The findings are in line with the bidirectional relationship between pain and sleep, explained by biological, psychological, and social factors (3, 5, 36), and additionally provide deeper insights into the lived experiences of those affected. In patients living with fibromyalgia or hypermobile Ehler-Danlos syndrome, insufficient sleep is one of the most challenging symptoms due to its extensive consequences (37, 38). Furthermore, pain and fragmented sleep are experienced as leading to more pain, fatigue, diminished activity during daytime and thus quality of life (25). Nevertheless, in our study, the participants descriptions of a vicious cycle, having difficulties in separating pain and sleep, are unforeseen. This finding is thus crucial to convey to healthcare professionals as they must be aware of the interconnection of pain and sleep and address the assessment and management of both conditions. Traditionally, pain rehabilitation programmes are based on a combination of psychological interventions, physical training and patient education performed by a multimodal team. Interventions in pain rehabilitation, including sleep, are performed to various extents and content; thus, evidence for its parts is not fully achieved yet (39, 40). Our findings stress the importance of addressing sleep health and sleep difficulties as mandatory when planning interventions for patients with chronic pain.

The comorbidity of sleep disorders, chronic pain and anxiety has previously been generally described (1, 26, 41). However, present findings add in-depth descriptions of how these experiences intervene in daily life and affect quality of life. The findings underscore the importance of identifying and treating sleep difficulties together with pain. For individuals with chronic musculoskeletal pain, a meta-analysis found that cognitive behavioural therapy for insomnia (CBT-I) significantly improved insomnia but did not affect pain (42), but CBT-I might not be available in all clinical contexts treating chronic pain patients, for example, in Sweden (43). In a Swedish clinical context, sleep education and hygiene are determined as a first step when addressing and treating sleep difficulties (44). The significance of sleep hygiene efforts for persons with chronic pain remains unclear (20, 45). In our study, the participants emphasised practical sleep aids, such as sliding sheets and pillows as beneficial to improving sleep and thus the pain experience. Furthermore, the benefits of the group, which enabled discussions of experiences and personal coping strategies, were expressed as significant, which is congruent with earlier research (46).

In contrast to a previous meta synthesis focusing on persons with fibromyalgia (25), our study includes a heterogeneous population with varied pain diagnoses. The prevalence of sleep disorders in patients with diabetic neuropathy was lower (11) compared to pain conditions observed in IPRP populations (47). The clinical experience is that patients with joint hypermobility are likely to face pronounced challenges in changing positions and finding comfortable postures when resting; hence, to the best of our knowledge, the sleep experiences of patients with joint hypermobility or neuropathic pain have barely been explored (48).

According to Antonovsky's salutogenic model, sense of coherence (49) is a global orientation where life is understood as more or less comprehensible, meaningful, and manageable. Sense of coherence refers to a person's ability to assess and understand a situation, find meaning to move in a health-promoting direction, and have the capacity to do so. Finding and experiencing meaning is an important factor for health in general and is also significant for patients living with chronic pain (50, 51). In this study, participants describe their striving for meaning and desire to balance and manage life with pain, sleep difficulties and fatigue. Issues like pacing, activity balance and resting were taught and discussed during present IPRP, which was experienced as helpful. This is in line with research where the role of these kinds of interventions is expressed as useful in different chronic conditions and pain (52, 53). The participants expressed a need for understanding, support, and self-compassion as cornerstones in rehabilitation changes, confirming findings from earlier studies on pain self-management interventions (54, 55).

### Strengths and limitations

Like all studies, this one has both strengths and limitations. The inclusion of participants with a broad range of pain diagnoses constituted a strength of the study, enriching the findings and providing insight into the diverse experiences and challenges faced by individuals with chronic pain. The heterogeneous sample in terms of diagnosis, age, and gender further strengthened the breadth of perspectives represented. However, diagnosis-specific differences in pain and sleep experiences, as well as potential differences between women and men, were not specifically explored and warrant further investigation.

The study's credibility was enhanced by using a pre-designed and tested topic guide as well as by having the same person conduct all the interviews. In terms of patient contribution and validation, the performed member check must be considered a strength as the participants confirmed the themes as recognisable. Thus, the findings may be interpreted as entailing some limitations as the participants were recruited from one single setting and IPRP. Additionally, there were some withdrawals during the sampling; however, this study population needs to be considered as a vulnerable group. Transferability was strengthened by presenting the context, participants demographics, and purposive sampling; however, the inclusion criteria of being able to speak Swedish may have introduced selection bias and limited the representation of individuals requiring interpreter support. While the experiences of the interplay between pain and sleep may be relevant across chronic pain populations, transferability to other rehabilitation settings and healthcare systems should be considered in relation to differences in healthcare organisation, rehabilitation models, and available support services.

## Conclusions

From a patient perspective, this study illustrates how chronic pain and sleep difficulties coexist, creating a vicious cycle difficult to distinguish, significantly impacting quality of life. The descriptions of practical, emotional, and social dimensions contribute to an extended understanding for individuals undergoing an interdisciplinary chronic pain rehabilitation programme (IPRP). The study concludes by emphasising the importance of incorporating sleep hygiene practices and sleep ergonomics, as well as adopting a holistic approach during IPRP.

## Data Availability

The raw data supporting the conclusions of this article will be made available by the authors, without undue reservation.
